# Predictors of successful vaginal birth after a cesarean section in Ethiopia: a systematic review and meta-analysis

**DOI:** 10.1186/s12884-023-05396-w

**Published:** 2023-01-26

**Authors:** Birye Dessalegn Mekonnen, Aragaw Awoke Asfaw

**Affiliations:** 1 Amhara Public Health Institute, Bahir Dar, Ethiopia; 2 Janamora Primary Hospital, Amhara Reginal State Health Bureau, Gondar, Ethiopia

**Keywords:** Vaginal birth after cesarean section, Systematic review

## Abstract

**Background:**

The rates of successful vaginal birth after previous cesarean section (VBAC) have been increasing with minimal complication. Successful vaginal birth after cesarean section improves maternal and fetal outcomes by shortening the length of hospital stay, avoiding abdominal surgery, decreasing the risk of infections and hemorrhage, and decreasing injury of the bladder and bowel. Despite a few single studies stating different predictors of successful VBAC, there is a lack of nationwide data to show the determinants of successful VBAC. Thus, this meta-analysis aimed to determine the predictors of successful VBAC in Ethiopia.

**Methods:**

A systematic literature search was performed from PubMed, Web of Sciences, EMBASE, CINAHL, and Google scholar until July 25, 2022. The quality of included studies was evaluated using the Joanna Briggs Institute (JBI) critical appraisal checklist. The analysis was executed using Stata 14 statistical software. Heterogeneity was evaluated statistically using Cochran’s Q-statistic and quantified by the I^2^ value. A random-effects model was used to estimate the determinants of successful vaginal birth after a cesarean section if substantial heterogeneity was detected across included studies; otherwise, a fixed-effects model was used.

**Results:**

Women living in rural residence (AOR: 2.14; 95% CI: 1.01, 4.52), history of previous spontaneous vaginal delivery (AOR: 2.92; 95% CI: 2.02, 4.23), previous successful vaginal birth after previous cesarean section (AOR: 5.29; 95% CI: 2.20, 12.69), history of stillbirth (AOR: 1.57; 95% CI: 1.20, 2.04), cervical dilation of ≥ 4 cm at admission (AOR: 2.14; 95% CI: 1.27, 3.61), spontaneous ruptured membranes at admission (AOR: 1.32; 95% CI: 1.17, 1.48) were independent determinants of successful vaginal birth after previous cesarean section.

**Conclusion:**

The results of this meta-analysis showed that successful VBAC was influenced by past and present obstetric conditions and other predictors. Thus, it is recommended that obstetric care providers should emphasize those factors that lead to successful vaginal birth during counseling and optimal selection of women for the trial of labour after cesarean section.

**Systematic review and meta-analysis registration:**

PROSPERO CRD42022329567.

**Supplementary Information:**

The online version contains supplementary material available at 10.1186/s12884-023-05396-w.

## Introduction

The World Health Organization (WHO) has reported that the cesarean section (CS) rate has increased in the world [1]. Cesarean section is significantly increasing though the WHO recommended the optimal rate of cesarean section to be between 5 and 15% [2]. Worldwide, about 21.1% of women gave birth by cesarean Sect. [3]. In Ethiopia, the prevalence of cesarean section among women who gave birth at health institutions was 29.55% [4].

Repeated cesarean section is associated with increased maternal complications, such as placenta previa, hysterectomy, adhesions, blood transfusions, and surgical injury [5]. Furthermore, the risk of postpartum death is higher in mothers who gave birth by cesarean Sect. [6, 7].

Despite cesarean section a life-saving medical procedure and intervention, the reasons for the continued increase are not completely understood [8, 9]. However, obstetricians’ uncertainty about the safety of the trial of vaginal birth after a previous caesarean section (VBAC), and unwilling of women to accept the trial of VBAC results in an increased cesarean delivery rate [10, 11]. Moreover, the decline in the rate of vaginal birth after cesarean section is the main reason for the rise in cesarean section rate [12, 13].

Vaginal birth after a previous caesarean section is an intended trial of vaginal birth by a woman who has had a previous cesarean Sect. [14]. Choosing the route of delivery after one previous cesarean section depends on the preferences of women, and past and present obstetric history [15, 16]. Candidates for a VBAC are women with low transverse hysterotomy, and singleton pregnancy with no contraindication for vaginal delivery [17, 18]. Careful selection of women for trial of labour after cesarean section delivery (TOLAC) remains a wise clinical decision as failed VBAC has the worst maternal outcomes [15, 19, 20].

Existing evidence have shown that the rates of successful VBAC among women with one cesarean section scar have been increasing with minimal complication [21–23]. Literature has also indicated that successful VBAC has improved maternal and fetal outcomes compared with delivery by repeated cesarean Sect. [24, 25]. Furthermore, a successful VBAC shortens the length of hospital stay, avoids abdominal surgery, decreases the risk of infections and hemorrhage, and decreases injury of the bladder and bowel [23, 26, 27].

The chance of vaginal birth after a previous cesarean section is determined by predictors such as maternal age, history of previous vaginal delivery, cervical dilatation at admission, rupture of membrane at admission, cephalo-pelvic disproportion, and birth weight > 4 kg [28–32]. Existing systematic review and meta-analysis shows that age, obesity, labor induction, birth weight, Bishop score, indications for the previous CS and previous vaginal birth were identified factors related to the success of VBAC [33].

Despite a few individual studies reporting different predictors of successful VBAC, there is a lack of nationwide data to show the determinants of successful VBAC. Thus, this meta-analysis aimed to determine the predictors of successful VBAC in Ethiopia. Accordingly, the population, intervention, comparator, outcome, study design (PICOS) framework was as follows: (P) Pregnant women, (I) trial of labour after cesarean section, (C) failed VBAC, (O) Determinants of successful VBAC among women with prior caesarean section.

## Methods

The study was conducted and reported following the Preferred Reporting Items for Systematic Reviews and Meta-Analyses (PRISMA) reporting guidelines (Supplementary file 1). The review was registered on the International Prospective Register of Systematic Reviews (PROSPERO) with the unique number CRD42022329567.

### Eligibility criteria

Observational studies involving women of child-bearing ages that were candidates for attempted vaginal birth with previous cesarean section in Ethiopia were considered. Articles that reported odds ratio (OR) with a 95% confidence interval (CI) for the binary factors in women with successful VBAC were included. Studies conducted only in Ethiopia, and published in or written in English were included. Whereas, studies that did not address determinants of successful vaginal birth after previous cesarean section were excluded. In addition, editorial reports, abstracts, letters, reviews, and commentaries were excluded from the study.

### Information sources and search strategy

A systematic literature search was performed from PubMed, Web of Sciences, EMBASE, CINAHL, and Google scholar until July 25, 2022. Medical Subject Headings, keywords, different boolean operators, and truncations were used in the search strategy. The following search keywords were used: "Vaginal Birth After Cesarean" OR "Vaginal birth*" OR "vaginal deliver*" OR "trial of labor*" OR "trial of labour" OR "active labor" OR "active labour" AND "Caesarean section" OR CS OR "abdominal deliver*" OR "uterine scar*" AND Determinant* OR Predictor* AND "Ethiopia". For instance, the search detail for PubMed Advanced search: ((((("Vaginal Birth After Cesarean"[All Fields] OR "vaginal birth*"[All Fields] OR "vaginal deliver*"[All Fields] OR "trial of labor*"[All Fields] OR "trial of labour"[All Fields] OR "active labor"[All Fields] OR "active labour"[All Fields]) AND "Caesarean section"[All Fields]) OR ("chemical synthesis"[MeSH Subheading] OR ("chemical"[All Fields] AND "synthesis"[All Fields]) OR "chemical synthesis"[All Fields] OR "cs"[All Fields]) OR "abdominal deliver*"[All Fields] OR "uterine scar*"[All Fields]) AND "determinant*"[All Fields]) OR "predictor*"[All Fields]) AND "Ethiopia"[All Fields]. Furthermore, bibliographies of selected articles were reviewed for additional potentially relevant studies. Moreover, Institutional Digital Libraries were searched to find grey literature.

### Outcome

The main outcomes of this meta-analysis were predictors of successful VBAC in Ethiopia. A successful VBAC is defined as spontaneous or instrumental assisted delivery to a woman undergoing a trial of labour after caesarean section delivery. In the meta-analysis, all of the pregnant women had experienced a trial of labour after a caesarean section.

### Study selection

All retrieved articles were exported to the EndNote X7.2.1 (Thomson Reuters, New York, USA) software citation manager to manage the screening process. Studies were initially reviewed based on their titles and abstracts. Then those studies deemed relevant were retrieved and reviewed in full text. The PRISMA 2020 flow diagram for new systematic reviews was used to summarize the study selection processes.

### Data extraction

Two reviewers (BDM and AAA) independently extracted pertinent data from each included article using a Microsoft Excel sheet. The following data were extracted from included articles: first author, year of publication, study setting, and design, sample size, and different determinants of successful VBAC.

### Assessment of risk of bias

The quality of included studies was evaluated by two reviewers (BDM and AAA) independently using the Joanna Briggs Institute (JBI) critical appraisal checklist adapted for case–control and cross-sectional studies. Any unclear information or disagreements between the two reviewers were resolved through discussion. Case–control studies were evaluated based on comparability and matching of groups, standard measurement, strategies to deal with cofounders, and statistical analysis, and rated from zero to ten-point scales. Cross-sectional studies were evaluated based on addressing the target population, adequacy of sample size, data collection methods, the definition of the variables, data collection tools, statistical analysis tests, study objectives, adequacy of response rate, and statistical analysis, and rated from zero to eight-point scales. Scores above five indicate low risk.

### Data synthesis

From each included study, the odds ratio (OR) for each factor with the 95% confidence intervals (CI) was extracted. Two reviewers (BDM and AAA) performed the data synthesis. We executed the effects of summary estimates with command “metan” using Stata 14.0 statistical program. Heterogeneity was evaluated statistically using Cochran’s Q-statistic and quantified by the I^2^ value. A random-effects model was used to estimate the determinants of successful vaginal birth after a cesarean section if substantial heterogeneity was detected across included studies; otherwise, a fixed-effects model was used. Publication bias was not evaluated because of the few numbers of studies for each factor.

## Results

### Study selection

**Fig. 1:**
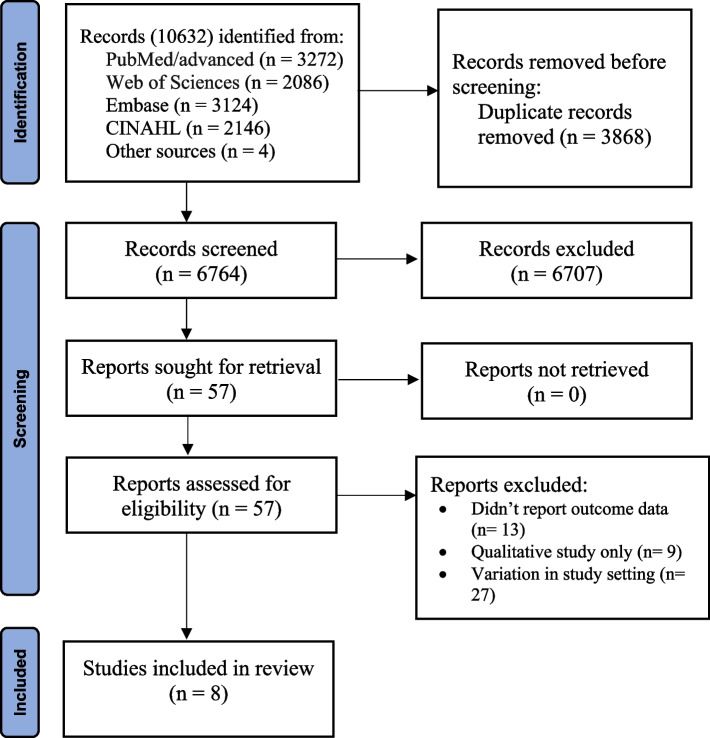
PRISMA flow diagram of study selection for the meta-analysis of predictors of VBAC in Ethiopia

A total of 10,632 records were identified from different databases. About 3,868 duplicate records were removed before the screening. Two reviewers (BDM and AAA) independently evaluated the remaining 6,764 records based on their titles and abstracts, which result in the further exclusion of 6,707 records. Additionally, 49 articles were excluded after reviewing 57 full texts articles. Lastly, a total of 8 studies were included in the final analysis (Fig. 1).

### Study characteristics

From a total of 8 studies were included, five were case–control studies, and three were cross-sectional studies. The sample size in the included studies ranged from 169 to 419. The Joanna Briggs Institute Critical Appraisal Checklist revealed a score of seven to eight in cross-sectional studies and seven to nine in case–control studies. In total, 2,100 women who attempted a vaginal birth after a cesarean section were included. Regarding geographical distribution, three of the studies were from Oromia [34–36], two from SNNPR [37, 38], two from Addis Ababa administrative city [39, 40], and one from Harari and Dire Dawa [41] (Table 1).

**Table 1:** Summary of studies included in the meta-analysis of predictors of successful vaginal birth after a cesarean section in Ethiopia, 2022

Author	Year	Region	Study area	Study design	Sample size	Quality	Determinants of successful vaginal birth after cesarean
Eyaya Misgan et al. [39]	2020	Addis Ababa	Addis Ababa	Cross-sectional	268	8	▪ Prior successful VBAC (OR 16.74; 95% CI 3.99–70.19) ▪ Women who had spontaneous ruptured membranes at admission (OR 2.67; 95% CI 1.28–5.57)
Birara and Gebrehiwot [40]	2013	Addis Ababa	Addis Ababa	Case–control	204	9	▪ No History of stillbirth (AOR: 2.54; 95% CI: 1.03,6.27) ▪ Prior successful VBAC (AOR: 3.40; 95% CI: 1.12, 9.48) ▪ Cervical dilatation at admission > 3 cm (AOR: 6.63; 95% CI: 3.36,13.01)
Mekonnin FT and Bulto. T [35]	2021	Oromia	Ambo town	Case–control	296	9	▪ Age less than 25 years (AOR: 8.88; 95% CI: 3.03, 26.03) ▪ History of previous successful VBAC (AOR: 3.01; 95% CI: 1.47, 6.13) ▪ History of previous spontaneous vaginal delivery (AOR: 3.85; 95% CI: 1.84, 8.05) ▪ Cervical dilation ≥ 4 cm at admission (AOR: 2.05: 95% CI: 1.14, 3.67)
Girma HT et al. [34]	2021	Oromia	Asella Referral Hospital	Case–control	288	7	▪ Rural residents (AOR: 2.419, 95% CI: 1.356, 4.316) ▪ History of any prior vaginal delivery (AOR: 3.723, 95% CI: 1.911, 7.254) ▪ Status of the membrane at admission (AOR: 2.349, 95% CI:1.287- 4.287) ▪ Prior successful vaginal birth after cesarean section (AOR: 15.471, 95%CI: 1.878–127.444)
Siraneh et al. [37]	2018	SNNPR	Gurage Zone	Cross-sectional	169	7	▪ Passage of liquor at admission (AOR: 0.25, 95% CI: 0.084,0.733) ▪ History of vaginal birth after cesarean (AOR: 1.88, 95% CI: 0.084, 0.733) ▪ Cervical dilation at admission (AOR: 8.171, 95% CI: 3.303, 34.473) ▪ Type of indication for previous cesarean section (AOR: 0.703, 95% CI: 0.014, 0.364)
Girma Y et al. [38]	2021	SNNPR	Mizan-Tepi University	Cros sectional	419	8	▪ Prior successful vaginal birth after cesarean section (AOR; 2, 95% CI: 1.18, 3.70) ▪ Previous successful spontaneous vaginal delivery (AOR; 4, 95% CI: 2.05, 7.83) ▪ Cervical dilatation at admission (AOR; 2.7, 95% CI: 1.47, 4.95) ▪ Duration of labor (AOR; 1.7, 95% CI: 1.07, 2.83)
Tefera M et al. [41]	2021	Harari and Dire Dawa	Eastern Ethiopia	Case–control	220	8	▪ Rural residents (AOR: 2.28; 95% CI: 1.85, 12.41) ▪ History of stillbirth (AOR: 0.07; 95% CI: 0.02, 0.53) ▪ Having ANC (AOR: 3.20; 95% CI: 1.15, 8.87) ▪ cervical dilatation of 6–8 cm at admission (AOR: 7.88; 95% CI: 2.17, 28.55) ▪ Following the women with partograph (AOR: 4.26; 95% CI: 1.90, 9.57)
Dereje L et al. [36]	2022	Oromia	East Wollega	Case–control	236	9	▪ Rural (AOR: 3.0, 95% CI: 1.25, 7.21) ▪ No history of stillbirth (AOR: 4.19, 95% CI: 1.20, 14.62) ▪ Prior VBAC (AOR: 2.4, 95% CI: 1.2, 6.4) ▪ Birth interval (AOR: 8.96, 95% CI: 3.25: 24.67)

### Determinants of successful vaginal birth after previous cesarean section

**Fig. 2:**
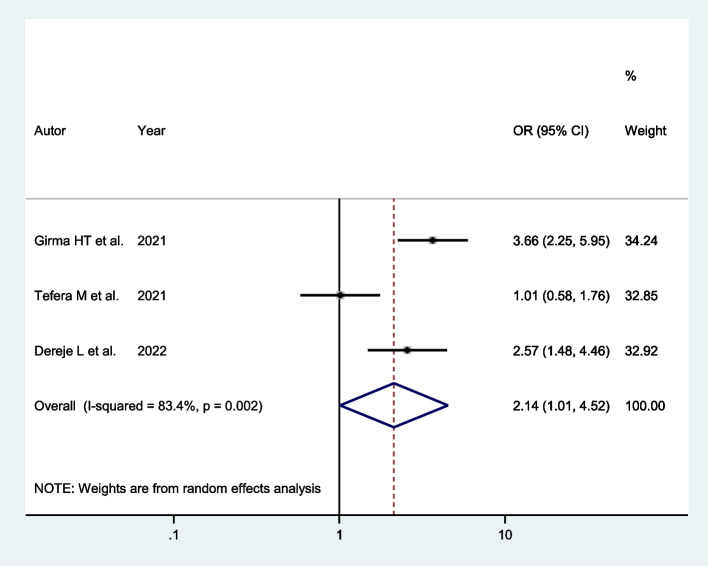
Forest plot of the association between residence and successful VBAC

Three studies indicated that women living in rural residence had a higher chance of successful VBAC. The pooled odds ratio indicated that the likelihood of successful VBAC was twice more likely among women living in rural residence (AOR: 2.14; 95% CI: 1.01, 4.52) than those women from urban residence (Fig. 2).

**Fig. 3:**
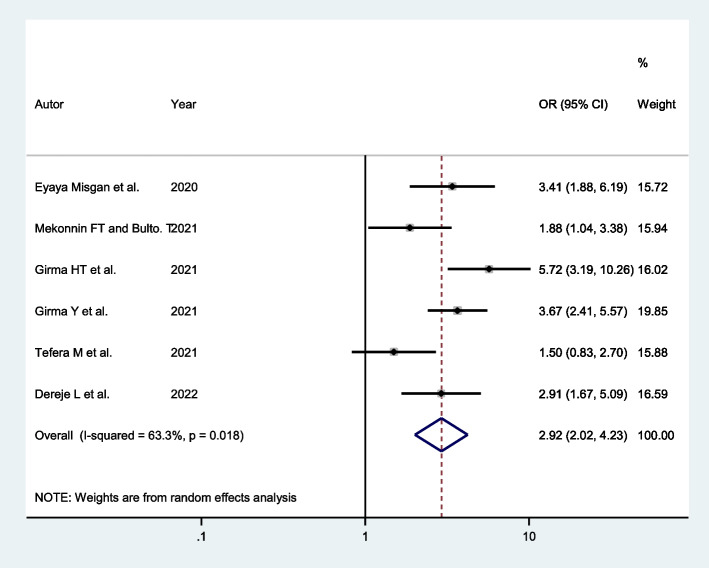
Forest plot of the association between a history of previous spontaneous vaginal delivery and successful VBAC

Six studies indicated that women with a history of previous spontaneous vaginal delivery had a higher chance of successful VBAC. The pooled odds ratio indicated that the odds of successful VBAC were almost three times higher among women with a history of previous spontaneous vaginal delivery (AOR: 2.92; 95% CI: 2.02, 4.23) compared to women who had no past spontaneous vaginal delivery (Fig. 3).

**Fig. 4:**
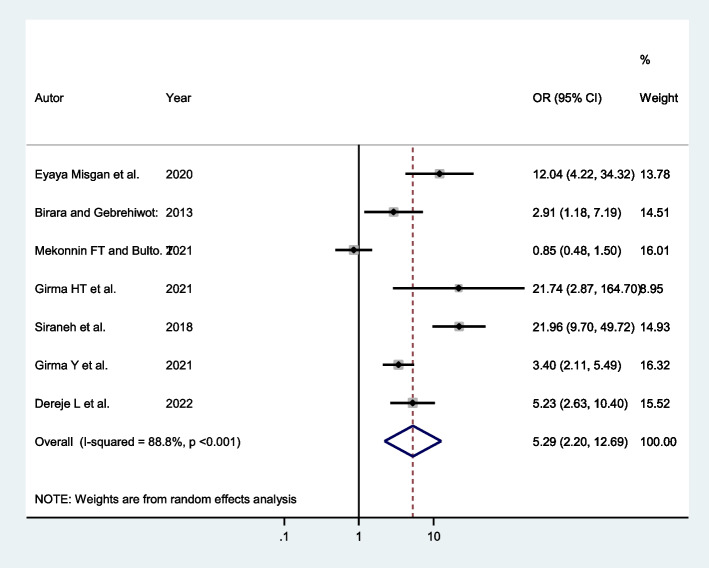
Forest plot showing the association between previous successful VBAC and successful VBAC

Seven studies indicated that women with previous successful VBAC had a higher chance of successful VBAC. Accordingly, mothers with previous successful VBAC have 5.29 times higher odds of having successful VBAC (AOR: 5.29; 95% CI: 2.20, 12.69) compared to their counterparts (Fig. 4).

**Fig. 5:**
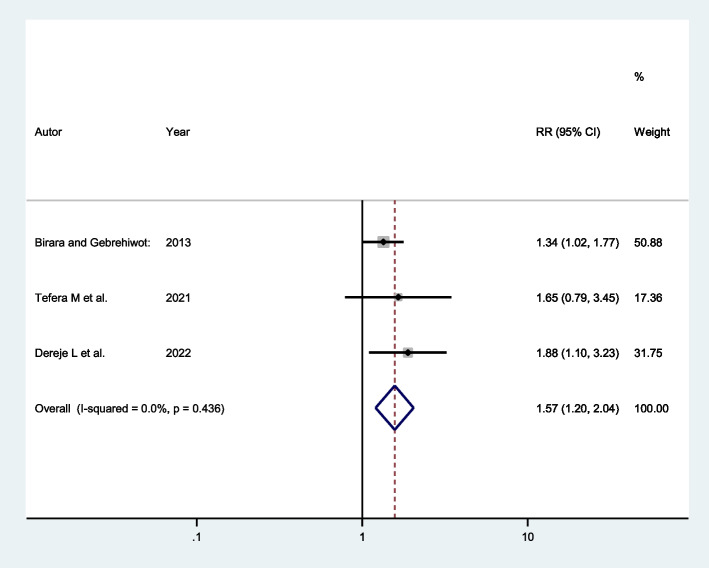
Forest plot showing the association between history of stillbirth and successful VBAC

Three studies indicated that women with a history of stillbirth had a higher chance of successful VBAC. The pooled odds ratio indicated that the likelihood of having successful VBAC was 1.57 times more likely among women with a history of stillbirth (AOR: 1.57; 95% CI: 1.20, 2.04) than those who had no history of stillbirth (Fig. 5).

**Fig. 6:**
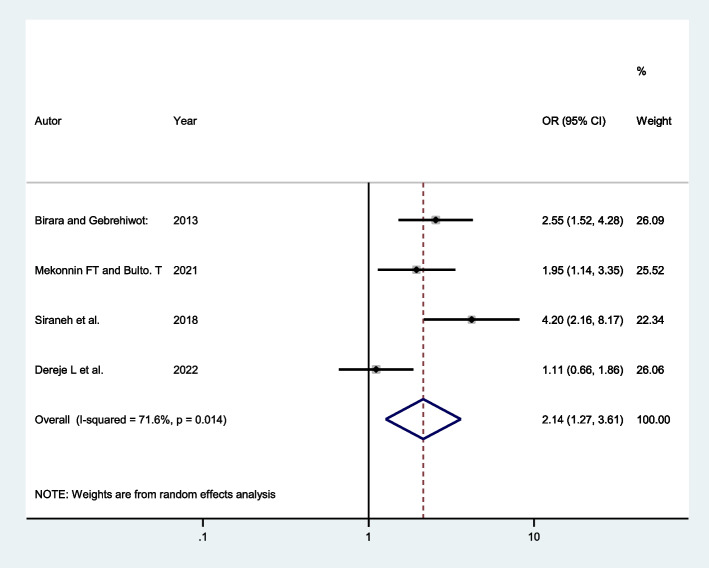
Forest plot showing the association between cervical dilation of ≥ 4 cm at admission and successful VBAC

Four studies indicated that women with cervical dilation of ≥ 4 cm at admission had a higher chance of successful VBAC. Accordingly, the pooled odds ratio indicated that the odds of having successful VBAC were twice more likely among mothers with cervical dilation of ≥ 4 cm at admission (AOR: 2.14; 95% CI: 1.27, 3.61) compared to those women with cervical dilation of < 4 cm at admission (Fig. 6).

**Fig. 7:**
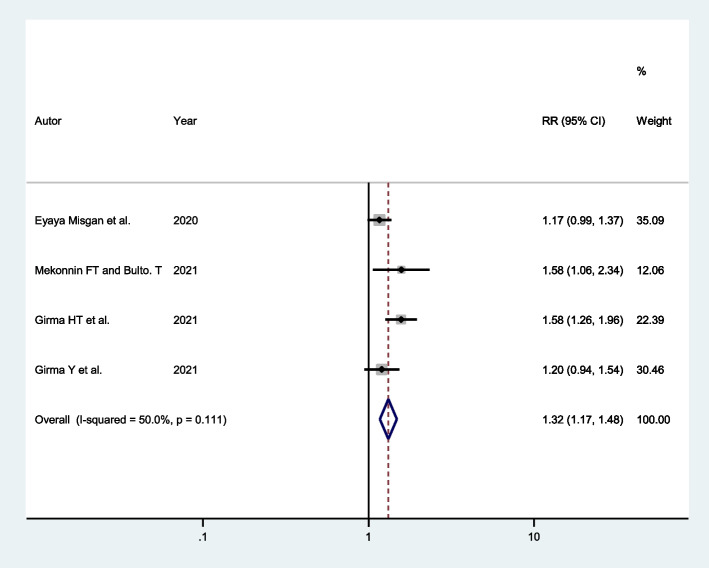
Forest plot showing the association between spontaneous ruptured membranes and successful VBAC

Four studies indicated that women who had spontaneous ruptured membranes at admission had a higher chance of successful VBAC. Accordingly, mothers who had spontaneous ruptured membranes at admission were 1.32 times more likely (AOR: 1.32; 95% CI: 1.17, 1.48) to have successful VBAC compared to mothers with intact membranes at admission (Fig. 7).

### Heterogeneity and publication bias and sensitivity analysis

Heterogeneity (I^2^ > 50%) was detected in the following meta-analysis: OR of rural residence, OR of history of previous spontaneous vaginal delivery, OR of previous successful birth after previous cesarean section, and OR of cervical dilation of ≥ 4 cm at admission. The rest two meta-analysis had ≤ 50% heterogeneity: OR of history of still birth, and OR of spontaneous ruptured membranes at admission. The number of included studies in each identified determinant of successful VBAC was limited. As a result, publication bias and sensitivity analysis were not evaluated in this meta-analysis.

## Discussion

The chance of VBAC for mothers varies based on obstetric and demographic characteristics [33, 42, 43]. This meta-analysis investigated the predictors of successful VBAC among women with prior cesarean section. Accordingly, rural residence, history of previous spontaneous vaginal delivery, previous successful VBAC, cervical dilation of ≥ 4 cm at admission, history of stillbirth, and spontaneous ruptured membranes at admission were significant determinants of successful VBAC. This implies that understanding the influencing predictors on VBAC could help to appraise chances for attaining a successful vaginal birth among women with prior CS.

Results of this meta-analysis indicated that mothers who live in rural residences were more likely to have successful VBAC than those who live in urban residences. The reason could be explained by the fact that women from rural residents would prefer vaginal birth because of fear of surgery and their lifestyles. This finding is supported by a study conducted in Turkey [44]. This meta-analysis also indicated that women with a history of stillbirth were more likely to have successful VBAC than those who had no history of stillbirth. This implies that having a history of poor pregnancy outcomes such as stillbirth can determine the mode of delivery [45]. Previous studies documented that adverse birth experiences have been identified as a strong predictor for a mode of delivery preference [46, 47].

This meta-analysis identified that women with a history of previous spontaneous vaginal delivery were more likely to have successful VBAC than those who had no past spontaneous vaginal delivery. This finding is supported by a previous systematic review [33]. The reason could be that multiparous mothers will develop effective uterine contractions in labour that will decrease subsequent problems [42]. Furthermore, previous vaginal delivery is associated with a reduced risk of uterine rupture, which further reduces the indication of CS [48]. Furthermore, mothers with previous successful VBAC were more likely to have successful VBAC compared to their counterparts. This could be attributed to the fact that a prior successful VBAC shortens the progress of labor and lowers the risk of subsequent rupture of the uterus [49]. Prior vaginal delivery following the prior CS was identified as a predictive indicator of successful VBAC [43].

This meta-analysis also identified that mothers with cervical dilation of ≥ 4 cm at admission were more likely to have successful VBAC compared to those with cervical dilation of < 4 cm at admission. The reason might be that mothers might be progressing to full dilation much more rapidly once they are in the active phase of labor, with faster progress of labor [50]. Evidence indicated that the prediction of the trial of labour after caesarean section delivery success was highly dependent on the initial cervical examination at the time of admission [33, 51]. Similarly, mothers who had spontaneous ruptured membranes at admission were more likely to have successful VBAC compared to those mothers with intact membranes at admission. The literature revealed that spontaneous ruptured membranes on admission were the most significant and strongest predictor for successful VBAC [52].

This review has the following potential limitations: the possible sources of heterogeneity were not addressed though heterogeneity between studies was exhibited. Furthermore, the studies included in this review represented only four regions of the country. Nevertheless, this meta-analysis provided the first quantitative evaluation of predictors of successful vaginal birth among women with prior cesarean section in Ethiopia, to the best of the authors’ knowledge.

This meta-analysis provides vital evidence to inform policy-makers, and other relevant stakeholders to understand the chances for achieving a successful vaginal birth among women with prior cesarean section. Some factors have been identified as being associated with an increased likelihood of successful VBAC. Hence, it could help healthcare providers to provide evidence-based counseling regarding VBAC, which has significant implications for avoiding repeated cesarean section.

## Conclusions

The results of this meta-analysis showed that living in rural residence, a history of previous spontaneous vaginal delivery, previous successful VBAC, cervical dilation of ≥ 4 cm at admission, a history of stillbirth, and spontaneous ruptured membranes at admission were factors significantly associated with successful VBAC. Therefore, hospitals and obstetric care providers should prepare a decision tool for the success of VBAC by considering past and present obstetric conditions and other predictors. It is also recommended that obstetric care providers should emphasize those factors that lead to a higher likelihood of successful vaginal birth during counseling and optimal selection of women for the trial of labour after caesarean section.

## Supplementary Information

**Additional file 1.** PRISMA 2020 checklist.

## Data Availability

All relevant data is included within the manuscript file.

## Abbreviations

AOR: Adjusted odds ratio
CBCS: Community-based cross-sectional
CI: Confidence intervals
JBI: Joanna Briggs Institute
OR: Odds ratio
VBAC: Vaginal birth after previous cesarean section
WHO: World Health Organization
